# ForePass outperforms Semaglutide in weight control, glucose metabolism, and gut microbiota in swine

**DOI:** 10.1111/dom.70167

**Published:** 2025-09-30

**Authors:** Sara Russo, Luca Proto, Manoel Galvao Neto, Giulia Angelini, Samantha Pezzica, Fabrizia Carli, Elena Previti, Maria Emiliana Caristo, Vincenzo Bove, Rima Chakaroun, Sara Roggiani, Valentina Tremaroli, Carel W. Le Roux, Stefan R. Bornstein, Amalia Gastaldelli, Ivo Boskoski, Geltrude Mingrone

**Affiliations:** ^1^ Institute of Translational Medicine and Surgery Università Cattolica del Sacro Cuore Rome Italy; ^2^ Bariatric Endoscopy Department of Mohak Bariatric and Robotic Center Sri Aurobindo Medical College Indore India; ^3^ Health Weight Loss and Bariatric Surgery Institute Orlando Health Orlando Florida USA; ^4^ Cardiometabolic Risk Laboratory, Institute of Clinical Physiology (IFC) National Research Council (CNR) Pisa Italy; ^5^ Department of Medical and Surgical Sciences Fondazione Policlinico Universitario A. Gemelli IRCCS Rome Italy; ^6^ Medical Department III—Endocrinology, Nephrology, Rheumatology University of Leipzig Medical Center Leipzig Germany; ^7^ Department of Pharmacy and Biotechnology University of Bologna Bologna Italy; ^8^ Department of Medical and Surgical Sciences University of Bologna Bologna Italy; ^9^ Wallenberg Laboratory for Cardiovascular and Metabolic Research, Department of Molecular and Clinical Medicine University of Gothenburg Gothenburg Sweden; ^10^ Diabetes Complications Research Centre University College Dublin Dublin Ireland; ^11^ Department of Internal Medicine III Carl Gustav Carus University Hospital Dresden, Technical University Dresden Dresden Germany; ^12^ Division of Diabetes & Nutritional Sciences, School of Cardiovascular and Metabolic Medicine & Sciences King's College London London UK

**Keywords:** animal pharmacology, antiobesity drug, GLP‐1 analogue, obesity therapy

## Abstract

**Aims:**

This study evaluated the metabolic efficacy of ForePass—a novel, incision‐free, reversible, endoscopically delivered device that mimics biliopancreatic diversion—in growing pigs. The primary aim was the superiority of ForePass over Semaglutide in improving insulin sensitivity (*S*_I_). Secondary aims included effects on weight gain, endogenous glucose production (EGP), disposition index (DI), oral glucose rate of appearance, plasma metabolomics, and faecal microbiota.

**Materials and Methods:**

Over 30 days, 12 young Landrace pigs (46.7 ± 1.1 kg) received ForePass, twice‐weekly Semaglutide, or sham endoscopy. Sample size was calculated a priori for the primary endpoint (Δ = 0.6 min^−1^·pM^−1^, SD = 0.3, *α* = 0.05, 80% power), yielding *n* = 4 per group. Body weight was monitored, and oral glucose tolerance testing (OGTT) with stable isotope tracers assessed hepatic glucose disposal. *S*_I_, insulin secretion, glucose rate of appearance (*R*_a_), metabolomics, and faecal microbiota were analysed.

**Results:**

ForePass improved S_I_ more than Semaglutide (2.75 ± 0.37 vs. 1.34 ± 0.21 min^−1^·pM^−1^) and sham (0.78 ± 0.46; *p* <0.05). Weight gain was 2.0 kg (4%) with ForePass, versus 16.3 kg (36%) with Semaglutide and 21.1 kg (47%) with sham (*p* <0.0001). Semaglutide reduced weight gain by 11% versus sham (*p* <0.05). DI was 2.6‐fold higher with ForePass than Semaglutide and 3.5‐fold higher than sham. ForePass reduced oral glucose *R*_a_ by 40% versus Semaglutide and 30% versus sham, while EGP 46% was lower than Semaglutide and 51% lower than sham (*p* <0.0001). Metabolomics showed ForePass increased ketogenic and branched‐chain amino acids, whereas Semaglutide raised lactate and alanine. Only ForePass increased faecal *Akkermansia muciniphila*.

**Conclusions:**

ForePass produced superior insulin sensitivity and weight outcomes versus Semaglutide. Its distinct effects on glucose disposal, metabolomics, and microbiota support development as a reversible, incision‐free endoscopic therapy that may bridge the gap between pharmacological and surgical options for obesity and type 2 diabetes.

## 1 INTRODUCTION

The emergence of new pharmacologic agents, particularly glucagon‐like peptide‐1 receptor agonists (GLP‐1 RAs), is transforming the therapeutic landscape for obesity and its related metabolic complications, in which insulin resistance represents a central pathogenic mechanism linking type 2 diabetes mellitus (T2DM), metabolic dysfunction‐associated steatotic liver disease (MASLD), and metabolic syndrome.

Randomised clinical trials have demonstrated that once‐weekly administration of 2.4 mg Semaglutide, combined with lifestyle modification, leads to a substantial reduction in body weight, averaging about 16% from baseline.^1^ These studies, however, primarily included participants with a mean body mass index (BMI) of 38 kg/m^2^,^1^, ^2^ thereby excluding most individuals with morbid obesity (BMI ≥40 kg/m^2^). For this population, bariatric surgery remains the principal therapeutic option, highlighting the unmet need for less invasive alternatives.

The ForePass device is a novel endoscopic procedure developed to mimic the metabolic benefits of bilio‐pancreatic diversion (BPD).^3^, ^4^, ^5^, ^6^ It integrates an intragastric balloon, which reduces gastric volume by approximately two‐thirds, with a central channel directing part of the ingested nutrients into a duodenal‐jejunal bypass sleeve that extends into the proximal jejunum, thus bypassing the foregut and delivering nutrients directly to the mid‐jejunum. This configuration is designed to induce weight loss and metabolic improvements through mechanisms similar to those of metabolic surgery.

Preclinical experiments in diet‐induced obese rats have shown that ForePass produces durable weight loss comparable to metabolic surgery and significantly enhances insulin sensitivity.^7^ Validation in larger animal models, however, is necessary to confirm these findings. Swine provide a particularly suitable translational model owing to their close anatomical, physiological, and genetic similarities to humans. In pigs, Semaglutide exhibits a pharmacokinetic profile with a half‐life of about 46.1 h following intravenous administration.^8^ Accordingly, we administered the drug twice weekly to maintain therapeutic plasma concentrations and ensure consistent pharmacodynamic action. Although this differs from the once‐weekly regimen used in humans, the approach accounts for interspecies differences in metabolism and provides a pharmacologically relevant schedule for the porcine model.

The aim of this study was to compare the effects of biweekly subcutaneous Semaglutide, at a per‐kilogram dose exceeding the approved clinical dosage, with those of the ForePass device and sham operation in Landrace pigs. Over a 1‐month treatment period, we evaluated changes in insulin sensitivity, body weight trajectory, plasma metabolomic profile, and gut microbiota composition. The primary endpoint was insulin sensitivity, reflecting its central role in the pathophysiology of obesity and T2DM. Weight gain was designated as the main secondary endpoint; although clinically important, it was considered subordinate, as improvements in insulin sensitivity provide a more direct mechanistic measure of metabolic benefit. Additional secondary endpoints included endogenous glucose production, disposition index, oral glucose rate of appearance, plasma metabolomics, and faecal microbiota composition.

By prioritising insulin sensitivity while also evaluating body weight, this study underscores the metabolic outcome most directly linked to disease mechanisms, while addressing the clinically relevant effect on weight regulation.

## 2 MATERIALS AND METHODS

### 2.1 Study design

#### 2.1.1 Sample size calculation

We planned a two‐sided comparison of mean insulin sensitivity (*S*_I_) between ForePass (*μ*_2_) and semaglutide (*μ*_1_), assuming independent groups with equal variance. Using *α* = 0.05 and power (1 − *β*) = 0.80, with expected means *μ*_2_ = 2.0 versus *μ*_1_ = 1.4 min^−1^·pM^−1^ and a common SD (*σ*) of 0.3, the detectable mean difference is Δ = 0.6 min^−1^·pM^−1^ (Cohen's *d* = Δ/SD = 2.0).

For a two‐sample *t*‐test:

$$\begin{array}{rcl} n_{\mathrm{per}\;\text{group}} & = & \frac{2\sigma^{2}{\left(z_{1-\alpha /2}+z_{1-\beta }\right)}^{2}}{{∆}^{2}}\\ & = & \frac{2{\left(0.3\right)}^{2}{\left(1.96+0.84\right)}^{2}}{{\left(0.6\right)}^{2}}=\frac{0.18\cdot 7.84}{0.36}=3.92 \end{array}$$

where *μ*_1_ and *μ*_2_ are the expected means for Semaglutide and ForePass, respectively. *σ* is the common standard deviation. Δ is the expected mean difference. *z*_(1 − *α*/2) = 1.96 is the standard normal deviate for a two‐sided test at *α* = 0.05. *z*_(1 − *β*) = 0.84 is the standard normal deviate for power = 0.80.

Rounding up the result, we require 4 pigs per group (total *N* = 8). We added a sham‐operated group as a control.

Experimental procedures were performed at the Animal House of the Catholic University in Rome, Italy, after approval from the Ethical Committee for animal studies. Twelve full‐sibling Landrace pigs (6 months old) were included in the study to minimise genetic variability. All animals were sourced from the same breeding facility and were matched for age and genetic background. We chose standard Landrace pigs to focus on the mechanistic effects of ForePass versus Semaglutide in a physiologically relevant large‐animal model. A unique identification number was assigned to each animal, and a standard diet (Crude protein 17.5%; crude fibre 3.9%; crude fat 3.5%; ash 5.2%) ad libitum was provided throughout the study. After 1 week of acclimation, pigs were randomly assigned to the Sham endoscopy (controls), the Forepass, or Semaglutide groups.

### 2.2 Semaglutide administration

To avoid vomiting, Semaglutide was administered subcutaneously twice weekly with a stepwise dosing regimen: 0.2 mg twice weekly during Days 1–7, followed by 0.4 mg twice weekly during Days 7–14, and finally 0.6 mg twice weekly during Days 15–30.

### 2.3 Endoscopic ForePass device implant and Sham endoscopy

**FIGURE 1:**
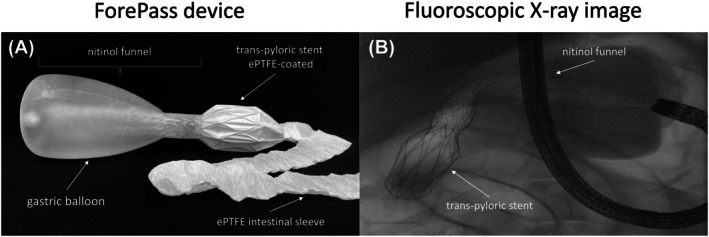
Design and anatomical positioning of the ForePass device in pigs. (A) Diagram of the ForePass device, consisting of a silicone gastric balloon attached to an expanded polytetrafluoroethylene (ePTFE) intestinal sleeve. A nitinol‐based funnel traverses the balloon and anchors the proximal end of the sleeve. To ensure stability within the gastrointestinal tract, a trans‐pyloric stent coated with ePTFE reinforces the device's position. (B) Fluoroscopic x‐ray image displaying the proximal components of the ForePass device in situ. The inflated gastric balloon, the nitinol anchoring structure within the duodenum, and the positioning endoscope are clearly visible.

The ForePass medical device (Keyron Ltd., London, UK) combines a silicone gastric balloon and a 60 cm expanded polytetrafluoroethylene (ePTFE) intestinal sleeve (Figure 1A). The balloon occupies 2/3 of the stomach volume (Figure 1B) and is connected to the sleeve through a nitinol stent‐like funnel. This design imitates gastric bypass by transporting food from the upper stomach to the intestines, reducing food intake and minimising contact with the upper gut mucosa.

The device was positioned endoscopically with gastric CO_2_ insufflation. All endoscopic procedures were performed under general anaesthesia and after an overnight fast. Anaesthesia was induced by intramuscular injection of Midazolam (0.5 mg/kg) coupled with ketamine (10 mg/kg), followed by endotracheal intubation. Anaesthesia was maintained by continuous intravenous infusion of diazepam (0.5 mg/kg) and ketamine (5 mg/kg), with pulse oximetry monitoring of heart rate and oxygen saturation.

The Sham endoscopy consisted of general anaesthesia, CO_2_ insufflation, and endoscopic manipulations of the same duration as for the ForePass implantation.

### 2.4 Oral glucose tolerance test

One month after the beginning of the study, an oral glucose tolerance test (OGTT) was performed under general anaesthesia. After an overnight fast, [6,6‐^2^H_2_]‐glucose was infused (priming dose: 32 μmol/kg; infusion rate: 0.32 μmol.kg^−1^.min^−1^) to measure endogenous glucose production. After 2 h of isotope infusion (basal period), 75 g of glucose, dissolved in saline solution and enriched with 0.9 g of [U^13^C]‐glucose tracer, was injected in the stomach using a gastric catheter positioned endoscopically. Samples for tracer enrichment, blood glucose, plasma insulin, and C‐peptide were taken at baseline, at 5, 10, 15, 20, and 25 min, and thereafter every 10 min until 180 min following the glucose load.

Plasma glucose concentrations were determined by the glucose oxidase method using a glucose analyser. Plasma insulin was measured by a porcine insulin ELISA (Mercodia, Uppsala, SE) with a detection limit of 1.15 (mU/L) and an intra‐ and inter‐assay precision of 3.5% and 4.3%, respectively. Plasma C‐peptide was measured by a porcine C‐peptide ELISA (Mercodia, Uppsala, SE) with a detection limit of 10 (pmol/L) and an intra‐ and inter‐assay precision of 3.6% and 3.8%, respectively.

### 2.5 Glucose and C‐peptide minimal models

Whole‐body insulin sensitivity from the OGTT was computed using the equations of the oral glucose minimal model^9^:

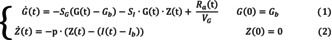




where G is glucose concentration ($G_{b}\;\text{baseline value}$), $Z\left(t\right)$ is the minimal model variable related to insulin action, $R_{a}\left(t\right)$ is the glucose rate of appearance when dealing with the OGTT, *I* is plasma insulin concentration and $I_{b}$ its baseline value.

$S_{G}$, $S_{I}$, *p*, $V_{G}$ are the estimated parameters in output of the glucose minimal model.

$S_{G}$ is fractional (i.e., per‐unit distribution volume) glucose effectiveness measuring glucose ability per se to promote glucose disposal and inhibit the net hepatic glucose balance, $S_{I}$ corresponds to insulin sensitivity, *p* is the rate constant of insulin action and $V_{G}$ represents the glucose distribution volume.

The profile of the insulin secretion rate (ISR) and the indexes of *β*‐cell sensitivity to glucose (the dynamic *β*‐cell glucose sensitivity, Φ_d_, the static sensitivity, Φ_s_, plus the total sensitivity (Φ)) were computed by the C‐peptide minimal model as proposed by Breda et al.^10^

Investigations^11^, ^12^ suggest that the parameters Φ_d_ and Φ_s_ have a cellular interpretation: Φ_d_ likely relates to exocytosis of insulin from secretory vesicles docked to the membrane and Φ_s_ reflects insulin granule translocation and maturation.^13^, ^14^ We have previously validated the minimal modelling in pigs.^15^

The area under the curve (AUC) of ISR was computed by the trapezoidal rule.

The disposition index (DI) was computed as Φ × *S*_I_.

The parameters of the glucose and C‐peptide minimal models were estimated by minimization of a weighted least‐squares index using a constrained Levenberg–Marquardt minimisation routine of the MATLAB library.

In these cases, a problem of parametric estimate occurs, that is, it needs to determine an estimate of the vector *θ* knowing the measures of glucose *z*_1_, *z*_2_, …, *z*_N_ and the output of the model *y*(*t*).

The goal is to find a way to reduce the prediction error *e*(θ) = *Z*–*Y* so that Euclidean norm *e*(*p*) is minimum.

This problem is resolved by finding an estimator that minimises the cost function:

(3) $$F\left(\theta \right)=\frac{\left[z-g\left(\theta \right)\right]\cdot W\cdot {\left[z-g\left(\theta \right)\right]}^{T}}{z\cdot z^{T}}$$

where *z* is the vector with blood samples, $g\left(\theta \right)$ is the output of the model (glucose or C‐peptide) in the same times of *z* and *W* a diagonal matrix containing weights.

To find the initial values of parameters, $\theta_{0}$, a fast‐greedy algorithm was implemented.

The standard errors of the estimates of individual parameters were evaluated by the linearization method, and the coefficients of variation were found to be <20%.

### 2.6 GC/MS analyses of stable isotopes

Isotopic enrichments of uniformly labelled glucose were measured by gas chromatography (GC)‐mass spectrometry (MS) (Agilent technology GC‐7890/MS‐5975; Santa Clara, CA, USA), equipped with a DB‐5 column (J&W, Agilent, Santa Clara CA, USA). For the analysis, 100 μL of plasma was deproteinized with 400 μL of methanol. Glucose was extracted using a modified Folch extraction procedure and measured after derivatization using acetic anhydride and pyridine, and enrichment was evaluated by monitoring ions at m/z 202/200 and 205/200 for [6,6‐^2^H_2_]‐glucose and [U^13^C]‐glucose, respectively.^16^ For all GC/MS analyses, instrument response was calibrated using standards of known enrichment.

### 2.7 Stable isotope calculations

The percent of ingested glucose that appeared in the systemic circulation was calculated from the plasma and OGTT glucose tracer‐to‐tracee ratio and rate of appearance (*R*_a_) values using Steele's equation as described previously.^17^ Total (endogenous and OGTT‐derived) glucose *R*_a_ into the systemic circulation, ingested glucose *R*_a_, and endogenous glucose *R*_a_ were calculated according to Reference [17]. Endogenous‐glucose‐production (EGP) was then computed as (total *R*_a_ − ingested *R*_a_).

### 2.8 Metabolomics

The metabolomic plasma profile was evaluated by GC/MS.^18^ Briefly, 60 μL of plasma with internal standards was deproteinized with 300 μL of methanol. Polar metabolites were extracted using the Folch extraction procedure.^19^ Amino acids and organic acids were measured by GC/MS/MS (Agilent Technology GC‐8890/MS‐7000GC/TQ) after methoxylation and derivatization using N‐methyl‐N‐(tert‐butyldimethylsilyl) trifluoroacetamide (MSTBSTFA, Sigma, USA).

For full quantification of selective organic acid, including lactate, TCA cycle metabolites, and ketoacids, we used the uniformly labelled ^13^C‐ internal standard mix MSK‐OA‐1 Labelled Organic Acid Mix (CIL Cambridge, MA, USA), while for amino acids quantification we used the uniformly labelled ^13^C‐ internal standard mix MSK‐A2‐S Metabolomics Amino Acid Mix standard (CIL Cambridge, MA, USA).

### 2.9 Metabolomics

The metabolomic plasma profile was evaluated by GC/MS.^18^ Briefly, 60 μL of plasma with internal standards was deproteinized with 300 μL of methanol. Polar metabolites were extracted using the Folch extraction procedure. Amino acids and organic acids were measured by GC/MS/MS (Agilent Technology GC‐8890/MS‐7000GC/TQ) after methoxylation and derivatization using N‐methyl‐N‐(tert‐butyldimethylsilyl) trifluoroacetamide (MSTBSTFA, Sigma, USA). Target metabolites were quantified using a labelled internal standard mix (MSK‐A2‐S Metabolomics Amino Acid Mix standard, CIL Cambridge, MA, USA and MSK‐OA‐1 Labelled Organic Acid Mix, CIL Cambridge, MA, USA).

### 2.10 Profiling of gut microbiota

Genomic DNA was extracted from 100 mg of faecal material using repeated bead beating as previously described.^15^ The V4 region of the 16S rRNA gene was amplified in duplicate 25 μL reactions containing 50 ng of template DNA, with 200 nM of the 515F and 806R primers,^20^ 0.4 mg/mL BSA, 5% dimethylsulfoxide, 0.15 μL of AccuPrime High Fidelity DNA Polymerase and Buffer II (Invitrogen). Thermocycling conditions consisted of an initial denaturation at 94°C for 2 min, followed by 25 cycles of denaturation at 94°C for 15 s, annealing at 52°C for 30 s, and elongation at 68°C for 30 s, with a final extension at 68°C for 2 min. PCR products were pooled per sample, purified using the NucleoSpin Gel and PCR Clean‐Up kit (Macherey‐Nagel), quantified with the Quant‐iT PicoGreen dsDNA assay (Invitrogen), normalised to 10 ng/μl, and pooled in equimolar concentrations. The final amplicon pool was purified using the AMPure XP Kit (Agencourt) and sequenced on the Illumina MiSeq platform (V2 chemistry; 2 × 250 bp paired‐ended reads).

Raw reads were merged, allowing up to five mismatches within an overlap of 251–257 bp. Reads with more than one expected error were excluded. Quality‐filtered reads were de‐duplicated and de‐noised using UNOISE3, and mapped as zero‐radius operational taxonomic units (zOTUs) using Usearch 11.0.067.^21^ Taxonomic classification of zOTUs was performed using DADA2's^22^ assignTaxonomy function (minBoot = 50) against the SILVA reference database v138.^23^ zOTUs representing <0.002% of total reads were discarded, and the remaining data were scaled to the total read count per sample prior to downstream analyses.

### 2.11 Statistics

All the data are expressed as means ± SEM unless otherwise specified. We used non‐parametric tests because many variables did not meet the assumption of normal distribution and because the sample size was small. The Friedman test was used for intragroup and the Kruskal–Wallis test for intergroup comparisons with Bonferroni adjustment for multiple comparisons. Statistical significance was set at *p* <0.05.

Glucose fluxes were calculated using non‐steady state equations as previously described.^18^

Glucose absorption time courses were available as group means for ForePass, Semaglutide, and Sham. To approximate subject‐level data, 12 simulated subjects per group were generated, assuming a coefficient of variation of 30% with multiplicative random noise, inducing within‐subject correlation. Group differences were expressed as ratios to Sham, analyzed on the log scale to align with a percent‐based non‐inferiority margin. Non‐inferiority was defined as the treatment/comparator ratio ≥0.80. One‐sided 95% confidence bounds were computed, and Holm's procedure was applied to adjust for the two simultaneous non‐inferiority tests.

Microbiota analyses were conducted in R (v4.2.2). zOTU‐level data handling and visualisation were performed using the phyloseq package (v1.42.0). Differences in community composition between groups were assessed using adonis2 (PERMANOVA) from the vegan package (v2.6.2)^24^ on weighted and unweighted UniFrac distances computed using the microViz R package (v0.12.7).^25^ Principal coordinates analysis (PCoA) was used for ordination, and group separation was evaluated via permutation testing (pseudo‐F statistic). Alpha diversity was assessed using Shannon diversity and observed zOTU richness with the microbiome R package (v1.30.0) (‘Microbiome’ n.d.), with group comparisons evaluated using the Wilcoxon rank‐sum test. Visualisations, including bar and box plots, were generated using ggplot2 (v3.5.2) and ggsignif (v0.6.4).

Differential abundance of taxa was tested using MetadeconfoundR (v1.0.2), with each treatment group compared individually to the others. Associations between genus‐level relative abundance and host weight, as well as metabolic parameters, were also assessed using MetadeconfoundR. Analyses were restricted to genera present at ≥0.2% relative abundance in at least two samples. Statistical significance was determined at a false discovery rate threshold of *Q* <0.1.

## 3 RESULTS

### 3.1 ForePass improves Insulin Sensitivity and reduces glucose and insulin responses more effectively than Semaglutide

**FIGURE 2:**
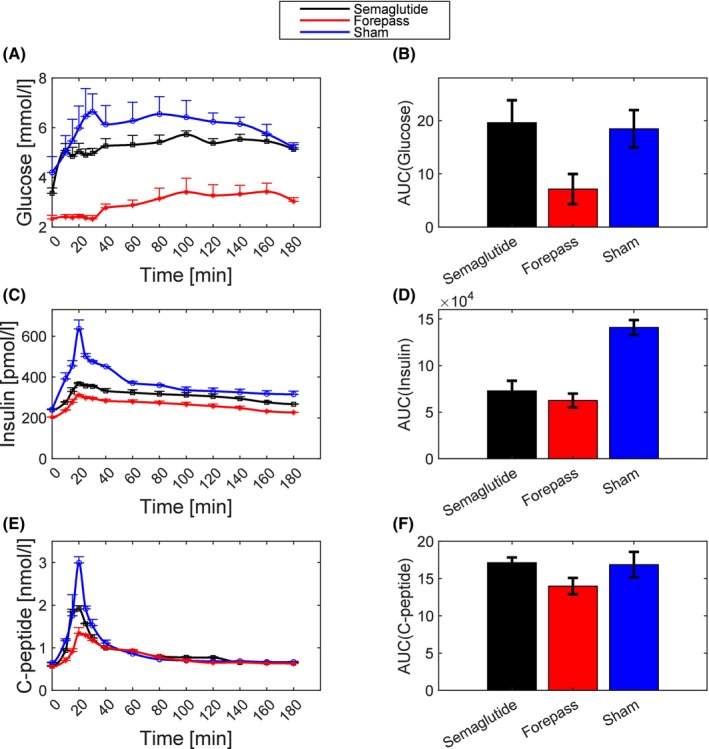
Postprandial glucose and insulin dynamics following ForePass treatment. Time‐course of (A) plasma glucose, (C) plasma insulin, and (E) plasma C‐peptide concentrations following OGTT in pigs treated with ForePass (*n* = 4), Semaglutide (*n* = 4), or Sham endoscopy (*n* = 4). Plasma glucose concentrations were significantly lower in the ForePass group compared with both Semaglutide and Sham. Plasma insulin concentrations were significantly lower in both ForePass and Semaglutide compared with Sham, with no difference between ForePass and Semaglutide. Few plasma C‐peptide concentrations were significantly lower in ForePass compared with Sham. Significant time points identified by repeated‐measures ANOVA followed by Tukey's HSD are provided in Supplementary Table 1. Panels show the corresponding AUCs for plasma glucose (B), insulin (D), and C‐peptide (F). Glucose AUC was significantly lower in the ForePass group compared with both Semaglutide and Sham (*p* <0.01). Insulin AUC was also reduced in ForePass and Semaglutide compared with Sham (*p* <0.05). Data are presented as mean ± SEM. Statistical comparisons were performed using repeated‐measures ANOVA with post hoc testing for the time‐course analyses and Kruskal–Wallis test with Bonferroni correction for AUC comparisons.

ForePass implantation led to profound improvements in glucose metabolism, exceeding those observed with Semaglutide. During the OGTT, the plasma glucose curve in ForePass‐treated pigs was nearly flat, indicating a striking reduction in postprandial glycaemic excursions (Figure 2). This effect is consistent with delayed gastric emptying and bypass of the duodenum and proximal jejunum induced by the ForePass device.^7^ The blunted glucose response was accompanied by only a modest increase in plasma insulin and C peptide levels, suggesting enhanced insulin sensitivity and a reduced need for insulin to maintain normoglycaemia (Figure 2).

#### 3.1.1 Insulin sensitivity

Insulin sensitivity (*S*_I_) was significantly lower in pigs treated with Semaglutide (1.34 ± 0.21 min^−1^·pM^−1^) and in those undergoing sham endoscopy (0.78 ± 0.46 min^−1^·pM^−1^) compared to ForePass‐treated animals (2.75 ± 0.37 min^−1^·pM^−1^), with both comparisons reaching statistical significance (*p* = 0.043). These findings indicate that ForePass markedly improved insulin sensitivity. Results from the minimal model analysis of the OGTT are summarised in Table 1.

**TABLE 1:** ‘Insulin Sensitivity and Secretion Data’.

	ForePass	Semaglutide	Sham	*P*	*P*	*P*
				ForePass versus Semaglutide	ForePass versus Sham	Semaglutide versus Sham
*S*_I_ × 10^4^ (min^−1^ × pM^−1^)	2.75 ± 0.37	1.34 ± 0.21	0.78 ± 0.46	0.043	0.043	NS
*S*_G_ × 10^2^ (min^−1^)	2.05 ± 0.51	1.42 ± 0.20	1.49 ± 0.43	NS	NS	NS
Φ_S_ × 10^9^ (min^−1^)	40.95 ± 6.96	32.39 ± 1.28	59.42 ± 9.78	NS	NS	NS
Φ_D_ × 10^9^	624 ± 111.04	746.53 ± 64.32	965.88 ± 23.29	NS	0.021	0.021
Φ_1_ × 10^9^ (min^−1^)	47.78 ± 5.73	39.49 ± 1.42	64.58 ± 8.45	NS	NS	0.043
AUC_ISR_ (nmol)	76.94 ± 6.49	86.96 ± 2.49	91.19 ± 2.89	NS	NS	NS

*Note*: *S*_I_ = insulin sensitivity; *S*_G_ = glucose effectiveness; Φ_S_ specifically evaluates the responsiveness of *β*‐cell insulin secretion to glucose, or *β*‐cell function, in a static condition, Φ_D_ specifically evaluates the responsiveness of *β*‐cell insulin secretion to glucose, or *β*‐cell function, in a dynamic condition; Φ_1_ parameter quantifies the first‐phase pancreatic beta‐cell's immediate response to the glucose challenge; AUC_ISR_ refers to the total insulin secretion over the duration of the minimal model test, calculated as the area under the curve (AUC) of the estimated insulin secretion rate (ISR). alpha = 0. 05. Between‐group differences were assessed using the Kruskal–Wallis test, with Bonferroni adjustment for multiple comparisons.

### 3.2 ForePass reduces weight gain more than Semaglutide

**FIGURE 3:**
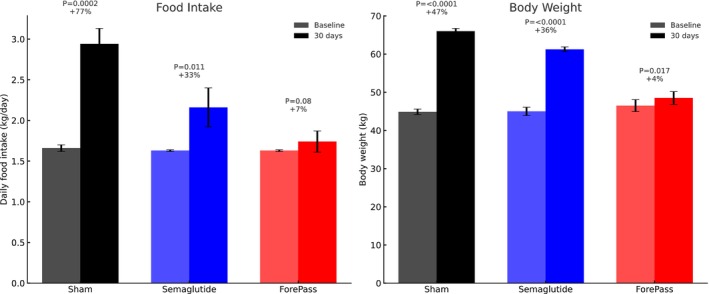
Effects of ForePass and Semaglutide on food intake and body weight gain in pigs. (A) Daily food intake at baseline and after 30 days in sham‐operated, Semaglutide‐treated, and ForePass‐treated pigs (*n* = 4 per group). Sham pigs exhibited a significant increase (*p* = 0.0002), Semaglutide‐treated pigs a modest increase (*p* = 0.011), while ForePass‐treated pigs showed no significant change (*p* = 0.08). Mean daily food intake over the treatment period was 26.5% lower in the Semaglutide group and 40.9% lower in the ForePass group compared with sham controls. (B) Body weight at baseline and after 30 days. Sham pigs gained 47% of body weight (*p* <0.0001), Semaglutide‐treated pigs gained 36% (*p* <0.0001), whereas ForePass‐treated pigs gained only 4% (*p* = 0.017). Data are presented as mean ± SD.

Throughout the study, all pigs were undergoing rapid physiological growth. After 30 days, animals implanted with ForePass exhibited a striking attenuation in weight gain compared to the other groups (Figure 3). Final body weight in the sham group increased from 44.88 ± 0.72 kg to 66.00 ± 0.71 kg (*p* <0.0001), reflecting a 47% gain. Semaglutide‐treated pigs gained less weight, rising from 45.00 ± 1.08 to 61.25 ± 0.63 kg (*p* <0.0001), a 36% increase. In contrast, ForePass‐treated pigs showed a minimal increase in body weight, from 46.5 ± 1.56 to 48.50 ± 1.67 kg (*p* = 0.017), corresponding to just a 4% gain. In other words, over the 30‐day period, ForePass‐treated pigs gained 32% less weight than those receiving Semaglutide and 43% less than sham controls. Semaglutide‐treated pigs also showed reduced weight gain, with an 11% decrease compared to the sham group.

This represents a highly significant reduction of weight gain compared with sham and Semaglutide, highlighting the superior effect of ForePass in suppressing excess weight accumulation during a period of active growth.

Sham pigs showed a marked increase in daily food intake, while the Semaglutide group had a modest rise and the ForePass group remained stable. Over the treatment period, average food intake was reduced by 26.5% with Semaglutide and 41% with ForePass compared to sham controls.

### 3.3 Oral glucose rate of appearance

Sham absorbed on average 3.11 ± 0.93 μmol/kg/min, ForePass absorbed 2.09 ± 0.63 μmol/kg/min, and Semaglutide absorbed 3.50 ± 1.05 μmol/kg/min. The ForePass/Sham ratio was 0.67, with a two‐sided 95% confidence interval of approximately 0.55–0.81. The Holm‐adjusted one‐sided lower bound was 0.55, which is below the non‐inferiority margin of 0.80, indicating that ForePass did not demonstrate non‐inferiority to Sham. The Semaglutide/Sham ratio was 1.13, with a two‐sided 95% confidence interval of approximately 0.93–1.37. The Holm‐adjusted one‐sided lower bound was 0.93, above the 0.80 margin, indicating that Semaglutide was non‐inferior to Sham. ForePass absorbed roughly one‐third less glucose than Sham. Therefore, ForePass absorbed ~30% less glucose than Sham and ~40% less than Semaglutide.

### 3.4 Insulin secretion

Total insulin secretion, measured as the area under the curve (${\mathrm{AUC}}_{\mathrm{ISR}}$) of insulin secretion during the OGTT, was 86.96 ± 2.49 nmol in Semaglutide‐treated pigs and 91.19 ± 2.89 nmol in sham‐operated animals, both of which were not significantly different from ForePass‐treated pigs (${\mathrm{AUC}}_{\mathrm{ISR}}$ = 76.94 ± 6.49 nmol). However, dynamic *β*‐cell glucose sensitivity (Φ_d_), an indicator of insulin secretory response to rising glucose, was significantly higher in the Semaglutide and sham groups compared with the ForePass group. Specifically, Φ_d_ values were 746.53 ± 64.32 and 965.88 ± 23.29 (×10^9^) in the Semaglutide and sham groups, respectively, versus 624.00 ± 111.04 (×10^9^) in the ForePass group (*p* = 0.021 for both comparisons vs. ForePass). Therefore, while Semaglutide, as expected, increased insulin secretion, ForePass did not, most likely because of reduced glucose absorption and improved insulin sensitivity.

This suggests that the increase in insulin secretion by β‐cells is primarily driven by dynamic adaptations in their glucose sensitivity, allowing them to compensate for reduced peripheral insulin sensitivity. Rather than a static increase in insulin output, *β*‐cells appear to modulate their responsiveness to glucose, enhancing insulin release in proportion to the prevailing level of insulin resistance.

#### 3.4.1 Disposition index

Pigs treated with Semaglutide (DI = 52.81 ± 8.02 pM^−1^·min^−2^) or subjected to sham endoscopy (DI = 39.13 ± 16.19 pM^−1^·min^−2^) exhibited significantly lower DI values compared to the ForePass group, which achieved the highest value driven by markedly enhanced insulin sensitivity (DI = 135.38 ± 29.48 pM^−1^·min^−2^; *p* = 0.043 vs. both Semaglutide and Sham).

### 3.5 ForePass more effectively suppresses endogenous glucose production than Semaglutide

EGP, a key determinant of fasting and postprandial glucose levels, was significantly reduced by both ForePass and Semaglutide compared to Sham controls. However, the magnitude of suppression was substantially greater with ForePass. Specifically, EGP in the ForePass group was reduced to 4.60 ± 0.39 μmol·pmol^−1^·min^−1^, representing more than a 50% reduction relative to Sham‐treated pigs (9.38 ± 1.16 μmol·pmol^−1^·min^−1^; *p* <0.0001). Semaglutide treatment also lowered EGP significantly (8.55 ± 2.28 μmol·pmol^−1^·min^−1^; *p* = 0.027 vs. Sham), but the effect was notably weaker than that achieved with ForePass (*p* <0.0001 for ForePass vs. Semaglutide).

These findings suggest that ForePass induces a more profound suppression of hepatic glucose output, likely due to enhanced insulin sensitivity, altered nutrient delivery, and reduced stimulation of intestinal gluconeogenesis.

### 3.6 The metabolomics profile is more favourable for ForePass than for Semaglutide

**FIGURE 4:**
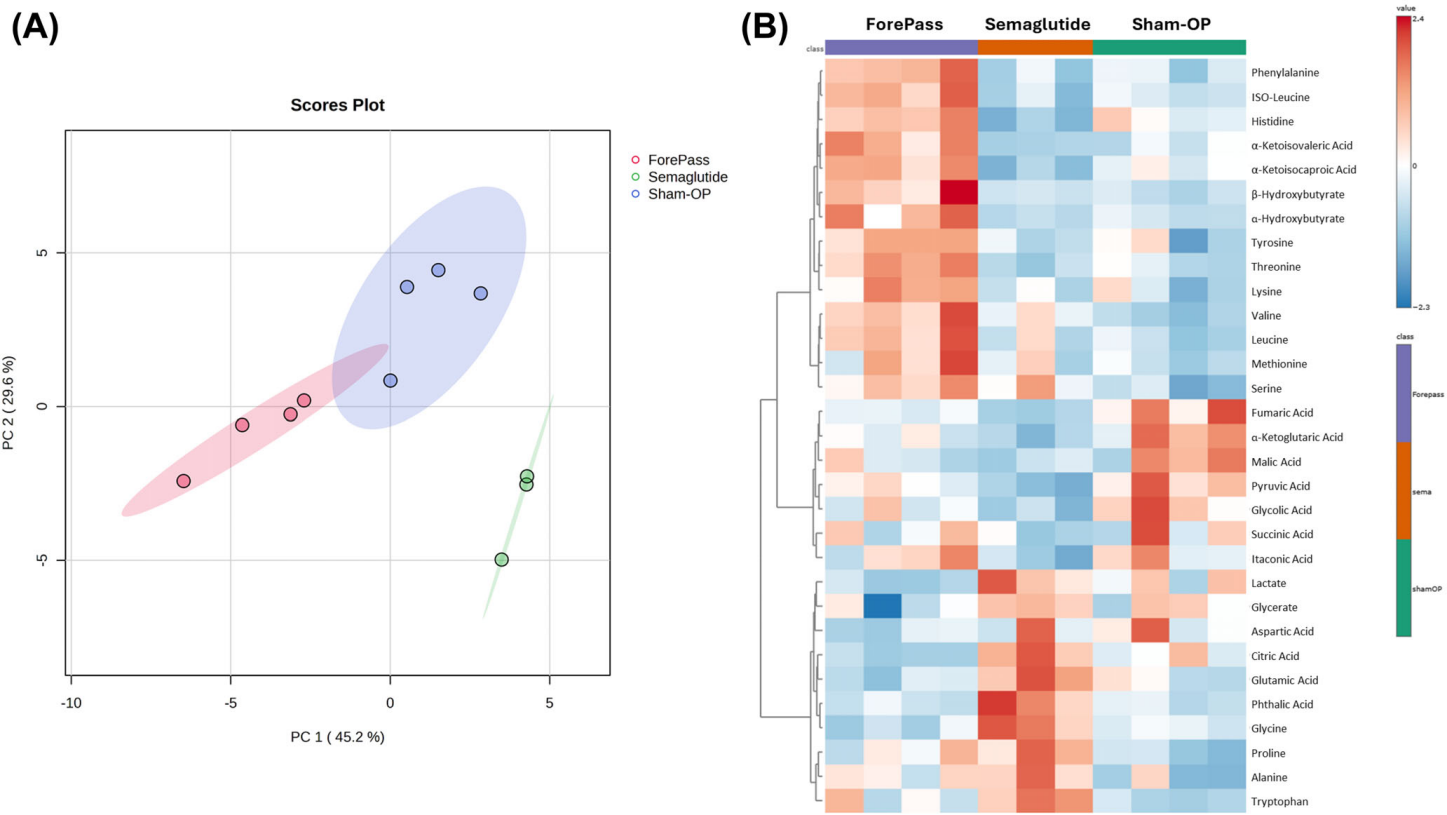
Metabolomic profiles. (A) Principal component analysis (PCA) of plasma metabolite data demonstrating clear separation among the ForePass, Semaglutide, and Sham‐operated groups, indicating distinct metabolic signatures. (B) Heatmap of unsupervised hierarchical clustering of plasma metabolite profiles across the same three groups, highlighting group‐specific patterns and metabolite‐level differences.

Untargeted metabolomic profiling, performed in 3 pigs rather than in 4 pigs in the Semaglutide group due to technical problems, revealed treatment‐specific alterations in circulating polar metabolites. PCA of plasma metabolite data showed a distinct separation between groups, indicating a global shift in systemic metabolism driven by the interventions (Figure 4A). ForePass‐, Semaglutide‐, and sham‐treated pigs each clustered separately, suggesting differential metabolic reprogramming depending on treatment modality.

In the ForePass group, 14 metabolites were significantly altered compared with Sham controls (Figure S1 in Supplementary Materials). Notably, ForePass was associated with increased plasma concentrations of several amino acids, including histidine, isoleucine, leucine, lysine, phenylalanine, serine, threonine, tyrosine, and valine, as well as alpha‐hydroxybutyrate, as highlighted in the heat map (Figure 4B). These changes may reflect a metabolic shift towards enhanced amino acid availability and changes in substrate utilisation.

In contrast, Semaglutide‐treated pigs exhibited higher plasma levels of alanine, glutamic acid, glycine, tryptophan, citric acid, and lactate compared with ForePass‐treated animals (Figure 4B), pointing to a distinct metabolic response, possibly associated with altered glycolytic and tricarboxylic acid cycle (TCA) cycle flux.

Lactate levels, fully quantified using U‐^13^C‐lactate as an internal standard, were significantly lower in the liver of the ForePass group compared with sham‐operated pigs (1985.66 vs. 3993.07 μmol/L; *p* = 0.016), suggesting reduced hepatic glycolytic flux or increased oxidative metabolism following duodenal/jejunal exclusion.

The observed changes in TCA intermediates align with the known metabolic flexibility of the TCA cycle. The cycle's intermediate pool is tightly regulated to maintain oxidative capacity across a wide range of energy states, including phases of active growth or caloric restriction. Despite reduced caloric intake with Semaglutide and ForePass, energy expenditure remained elevated due to the physiological demands of growth, suggesting the activation of compensatory metabolic mechanisms to sustain energy balance.

Prior studies^26^ have shown that leucine flux remains stable during short‐term caloric restriction when protein intake is preserved. In our study, despite reduced energy intake, protein intake was maintained at approximately 1.2 g/kg/day, which may have supported amino acid availability while shifting energy metabolism towards alternate fuels.

### 3.7 Gut microbial diversity and composition are differentially affected by Forepass and Semaglutide

To assess the impact of ForePass and Semaglutide treatments on gut microbial ecology, we profiled the V4 region of the 16S rRNA gene in faecal samples.

**FIGURE 5:**
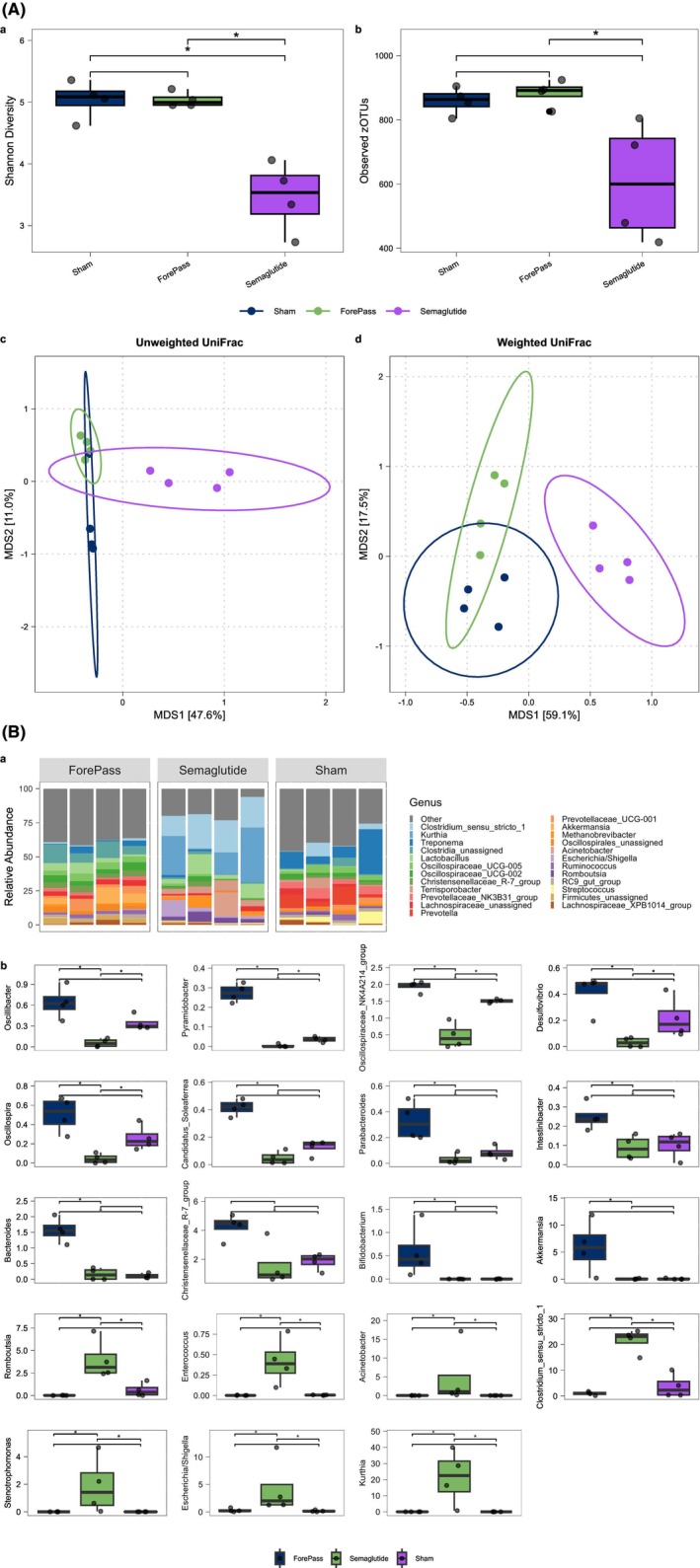
(A) Gut microbiome diversity and composition in Sham, ForePass, and Semaglutide groups. Alpha diversity estimated using Shannon diversity (a) and observed zOTUs (b). Pairwise Wilcoxon. *p ≤0.05 principal coordinates analysis (PCoA) of beta diversity based on zOTUs, calculated using weighted UniFrac (c) and unweighted UniFrac distances (d). (B) Differentially abundant taxa in Sham, ForePass, and Semaglutide groups. (a) Stacked bar plots showing the relative abundance of the top 25 bacterial genera in the gut microbiota of the three experimental groups. *n* = 4 pigs per group. (b) Relative abundance of selected bacterial genera across experimental groups (enriched taxa in both ForePass and Semaglutide, specified up to genus level). Pairwise Wilcoxon. *p ≤0.05.

ForePass‐treated animals showed no significant changes in alpha diversity relative to Sham controls (Wilcoxon rank‐sum; *p* = 0.7 for Shannon index; *p* = 0.5 for observed zOTUs), while Semaglutide significantly reduced alpha diversity compared to both Sham and ForePass (Wilcoxon rank‐sum; Shannon index: Sham vs. Semaglutide *p* = 0.03, ForePass vs. Semaglutide *p* = 0.03; observed zOTUs: Sham vs. Semaglutide *p* = 0.06 and ForePass vs. Semaglutide *p* = 0.03) (Figure 5A,a,b). These results suggest that Semaglutide had a more substantial impact on the gut ecosystem, leading to more constrained and less diverse microbial communities in the pigs.

Consistent with the reduced alpha diversity, beta diversity analyses revealed marked compositional shifts between treatment groups as measured by both weighted UniFrac and unweighted UniFrac distances (PERMANOVA; *R* ^2^ = 0.68, *p* = 0.0003 and *R* ^2^ = 0.51, *p* = 0.0004, for weighted and unweighted UniFrac, respectively; Figure 5A,c,d). PCoA showed that the Semaglutide group was separated from both the Sham and Forepass groups along the first axis. In contrast, the second axis, explaining a smaller amount of compositional variance, separated Sham animals from those receiving either ForePass or Semaglutide. The separation was most pronounced using weighted UniFrac, suggesting that differences in relative abundance, rather than the mere presence/absence of taxa, underlie the observed results.

### 3.8 ForePass and Semaglutide influence the relative abundance of different taxa linked to metabolic parameters

Analysis of differentially abundant taxa in ForePass and Semaglutide treatment groups compared to controls showed that ForePass and Semaglutide influenced the abundance of distinct taxa (Figure 5A,a,b). The ForePass group exhibited an increased relative abundance of several taxa associated with metabolic health, including *Christensenella*, *Bifidobacterium*, and *Intestinibacter*, as well as *Ruminococcus* and multiple members of the *Oscillospiraceae* family (Figure 5A,b). In contrast, in Semaglutide‐treated pigs, many genera with relatively high abundance in Sham and ForePass were substantially depleted, while few taxa from Firmicutes and Proteobacteria bloomed. These included *Clostridium* sensu stricto *1*, *Kurthia*, *Escherichia‐Shigella*, *Enterococcus*, *Romboutsia*, *Acinetobacter*, and *Stenotrophomonas* (Figure 5A,b).

Taxa increased in ForePass, such as *Akkermansia* and *Bacteroides*, correlated significantly (*p* <0.05) with improved glycemia outcomes, including reduced glucose AUC and lower fasting insulin levels. However, the taxa that increased or decreased after Semaglutide treatment did not associate with the metabolic effects of the treatment, thus reinforcing the link between ForePass and a metabolically favorable microbial signature.

**FIGURE 6:**
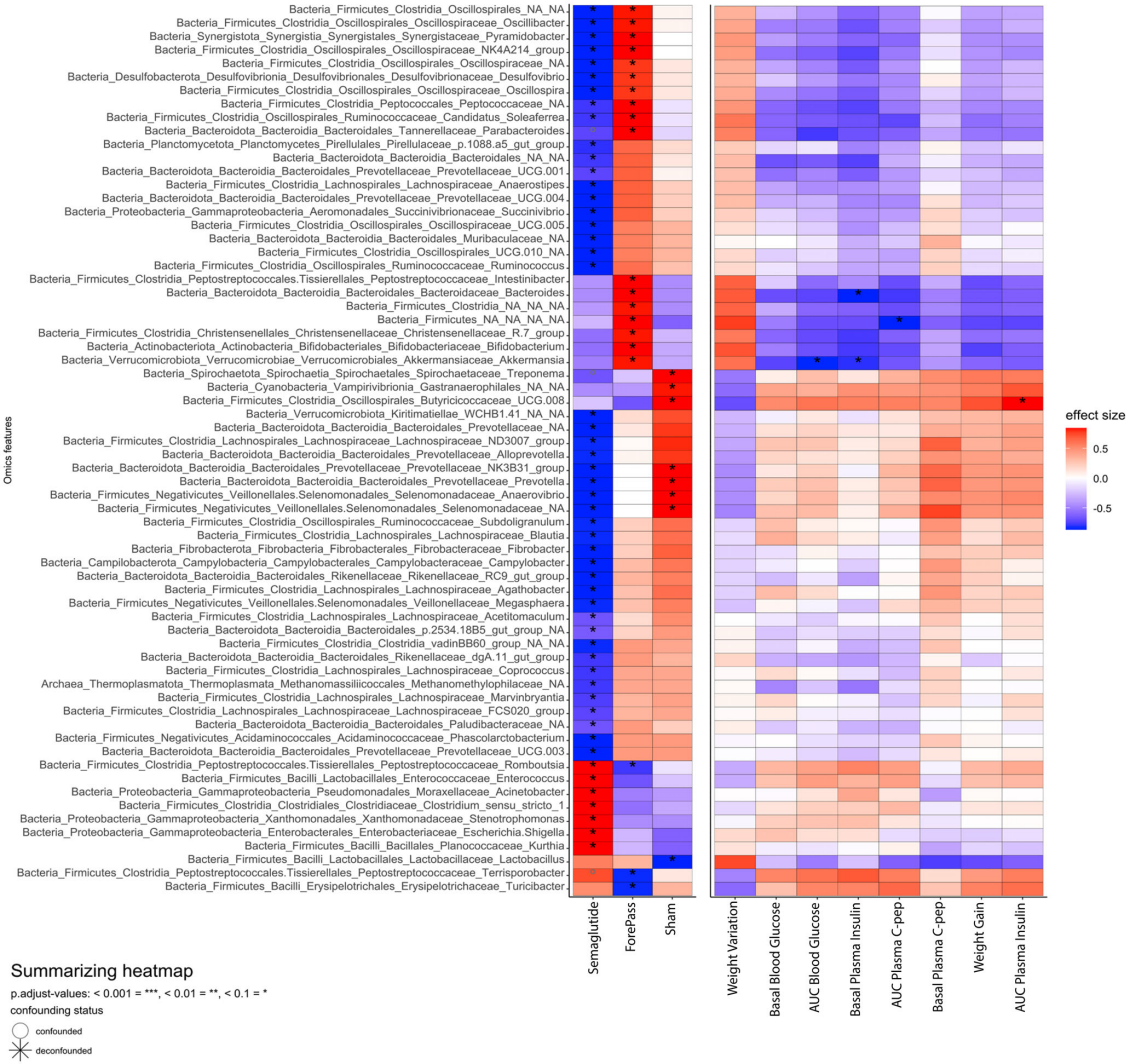
Associations with host metabolic parameters for genera differentially abundant in ForePass and Semaglutide groups compared to controls. Heatmap displaying correlations between the relative abundance of genera and treatment (Sham, ForePass, or Semaglutide), body weight, and metabolic parameters. Only genera with a relative abundance >0.2% in at least two samples are shown. The red–blue colour scale represents effect sizes, with red indicating positive and blue indicating negative correlations. *n* = 4 pigs per group. *Q* <0.1.

Our results (Figure 6) also suggest that the decrease in community complexity following Semaglutide treatment might favor stress‐tolerant and opportunistic taxa (such as *Escherichia–Shigella, Enterococcus*, and *Acinetobacter*), potentially reflecting shifts in nutrient gradients and gut motility.

## 4 DISCUSSION

The ForePass device is a novel endoscopic approach that reduces gastric volume by about two‐thirds while diverting nutrients past the duodenum and proximal jejunum, thereby reproducing some of the mechanisms of BPD. BPD is among the most effective metabolic surgeries, producing diabetes remission and substantial weight loss,^3^, ^4^, ^5^, ^6^ and is currently indicated for patients with BMI ≥40 kg/m^2^ or ≥35 kg/m^2^ in the presence of comorbidities.^27^

In our study, ForePass surpassed Semaglutide in improving insulin sensitivity, attenuating weight gain, and inducing favourable metabolomic and microbiota changes in growing pigs. Semaglutide was administered biweekly at a per‐kilogram dose higher than the approved clinical regimen, based on pharmacokinetic data in swine.^8^ Despite this intensified schedule, its effects remained less pronounced than those of ForePass. The device not only slowed gastric emptying, as previously shown in rodents,^7^ but also diverted nutrients away from the foregut, thereby reducing carbohydrate absorption. Consistently, the rate of glucose appearance following oral gavage was about 30% lower with ForePass than with sham or Semaglutide.

The limitations of pharmacological therapy underscore the value of alternative strategies. Current ADA and EASD guidelines recommend maintaining HbA1c ≤7% (≤53 mmol/mol) to minimise diabetes complications,^28^ yet only 40%–54% of patients achieve this goal.^29^, ^30^ Even with GLP‐1 receptor agonists, two‐thirds of patients fail to reach HbA1c <7% after 6 months, and fewer than half achieve ≥5% weight loss, with only a quarter losing ≥10%.^31^ Moreover, Semaglutide's weight‐reducing effect is halved in individuals with type 2 diabetes.^32^ Tirzepatide achieves greater weight loss but remains less effective in diabetic than in non‐diabetic patients.^33^, ^34^ These observations highlight the need for interventions that act beyond weight reduction to improve insulin sensitivity and glucose control.

The duodenum has emerged as a central regulator of metabolic homeostasis, influencing appetite, gastric emptying, hepatic glucose output, and islet hormone secretion through nutrient sensing and neurohormonal pathways.^35^ Diet‐induced villus hyperplasia, as seen in obese and prediabetic mice, enhances nutrient absorption while impairing enteroendocrine regulation.^36^ In our model, ForePass directly addressed these mechanisms: it not only limited weight gain but also improved insulin sensitivity more robustly than Semaglutide. Consistent with human data from the SUSTAIN 1–3 trials, the modest effect of Semaglutide on insulin resistance was largely mediated by weight reduction.^37^ ForePass, by contrast, combined weight attenuation with exclusion of the duodenum and proximal jejunum, targeting glucose absorption and insulin resistance through mechanisms resembling BPD.^6^

Metabolic profiling further underscored these differences. ForePass animals exhibited significantly lower hepatic lactate levels, suggesting enhanced oxidative metabolism. Because the liver clears about 70% of systemic lactate,^38^ elevated concentrations usually reflect reduced oxidation of lactate to pyruvate and diminished TCA cycle entry. During rapid growth, when energy demand is high, ForePass pigs with lower intake appeared to shift towards ketone and amino acid metabolism for ATP generation (Figure S2). This interpretation was supported by lower insulin levels during OGTT and higher circulating ketone bodies, pointing to increased lipolysis and reliance on fat‐derived fuels. Such adaptations favor glucose sparing and prioritise ketones for energy‐intensive tissues. Reduced protein oxidation may also have contributed, possibly through downregulation of branched‐chain 2‐oxoacid dehydrogenase, the rate‐limiting enzyme in BCAA catabolism, thereby preserving circulating amino acids.

By contrast, Semaglutide was associated with higher lactate and alanine flux into pyruvate, while aspartate contributed via oxaloacetate. Other substrates, including glycine, proline, and tryptophan, fed into gluconeogenic or redox pathways and the kynurenine pathway, consistent with a distinct metabolic strategy (Figure S2).

Gut microbiota composition also diverged between groups. Semaglutide reduced microbial diversity and enriched stress‐tolerant and opportunistic genera such as *Escherichia*–*Shigella*, *Enterococcus*, and *Acinetobacte*r.^39^ ForePass, in contrast, preserved microbial richness and increased taxa linked to metabolic health. These findings parallel rodent studies in which liraglutide or dual GLP‐1/GLP‐2 agonists reduced diversity and favored low‐abundance, inflammation‐associated taxa.^40^ Notably, Akkermansia muciniphila, enriched after Semaglutide in rodents and humans,^41^, ^42^ was increased only in ForePass pigs. Given its role in mucosal integrity and hepatic gluconeogenesis, Akkermansia may explain the greater suppression of endogenous glucose production and stronger improvement DI with ForePass.

Such differences likely reflect species‐specific features of gut ecology. Although pigs and humans share many physiological and anatomical traits, such as a comparable ratio of intestinal length to body size, they diverge in others, including a prominent gastric diverticulum and a spiral colon, which create unique microbial niches.^43^, ^44^ Rodents differ even more markedly, limiting their translational value.^45^ Our results therefore suggest that pigs capture clinically relevant ecological effects of Semaglutide more accurately than rodent models.

This study has several limitations: the intervention period was short, outcomes reflected attenuated weight gain rather than weight loss, metabolomic profiling included only three Semaglutide animals, and the absence of baseline microbiota profiles limits causal inference for microbial shifts. However, the findings are robust, utilising a thorough, multi‐modal approach that included randomised group assignment with a sham control, standardised husbandry and diet, isotope‐tracer OGTT with non–steady‐state modelling to quantify glucose fluxes, a clearly defined primary endpoint (insulin sensitivity) corroborated by EGP suppression, DI, and glucose appearance AUC, and parallel metabolomic and 16S rRNA profiling analysed with appropriate statistics. The consistency and magnitude of effects across these independent modalities strengthen the overall conclusions despite the noted constraints.

From a translational perspective, ForePass may be particularly valuable for patients with obesity complicated by insulin resistance and type 2 diabetes. In these individuals, the device reduced intestinal glucose absorption and hepatic glucose production, addressing both peripheral and hepatic insulin resistance—features often inadequately targeted by incretin‐based therapies. ForePass could also benefit patients with progressive weight gain despite pharmacotherapy or those unable to tolerate GLP‐1 receptor agonists. Its minimally invasive and reversible nature further supports use in individuals at high surgical risk or unwilling to undergo bariatric surgery.

In conclusion, ForePass demonstrated superior efficacy compared with Semaglutide in improving insulin sensitivity, limiting weight gain, and favourably modifying metabolic and microbial profiles. While Semaglutide was more effective than Sham, its impact was substantially lower than that of ForePass. These findings support further clinical evaluation of ForePass as a reversible, incision‐free endoscopic therapy that may bridge the gap between pharmacological and surgical options for obesity and type 2 diabetes.

## CONFLICT OF INTEREST STATEMENT

IB reports consulting fees from Apollo, Endosurgery, AndoTools, and Nitinotes. G.M. has received consulting fees from Novo Nordisk, Eli Lilly, Boehringer Ingelheim, Johnson and Johnson, Medtronic, Fractyl Inc., and Recor Inc. She also serves as a Scientific Advisor for Keyron Ltd., Metadeq Inc., GHP Scientific Ltd., and Jemyll Ltd. G.A. has received consulting fees from Metadeq Inc. F.R. has received research grants from Ethicon and Medtronic; consulting fees from Novo Nordisk, Ethicon, and Medtronic; and serves on scientific advisory boards for GI Dynamics and Keyron. VT is the co‐founder and shareholder of Roxbiosens Inc. Erbe Elektromedizin, Boston Scientific, Cook Medical, and Pentax Medical.

MGN reports consulting fees from Apollo EndoSurgery, USGI, and Keyron. He is also a Scientific Advisor of Keyron and Morphic Medical.

VB reports consulting fees from Apollo EndoSurgery. Other authors declare no competing interests. Keyron Ltd. had no role in the study design, data collection, data analysis, or writing of the manuscript. The data are available upon reasonable request.

## PEER REVIEW

The peer review history for this article is available at https://www.webofscience.com/api/gateway/wos/peer-review/10.1111/dom.70167.

## Supporting information


**Data S1:** Supporting Information

## Data Availability

Single data for insulin sensitivity and secretion are provided in an excel file in the Supplements.Other data are available upon reasonable request.
