# Supplementary material for: ForePass outperforms Semaglutide in weight control, glucose metabolism, and gut microbiota in swine
Source: Diabetes Obes Metab. 2025 Sep 30;27(12):7587–601. doi: 10.1111/dom.70167 (PMC12587247; doi:10.1111/dom.70167)
Supplement: Supplementary file 1 — Data S1: Supporting Information [file DOM-27-7587-s001.zip › dom-25-2790-op-File009.docx]

**Figure 1S**

**Changes in circulating metabolites after ForePass Device implantation (ForePass) and Semaglutide treatment (Semaglutide) vs Sham-operation (Sham-op) in the fasting state**. A general increase in the concentrations of alpha-ketoacids, beta-hydroxybutyrate, and amino acids (BCAA and AAA) was observed in the ForePass group compared to Sham-OP. In contrast, Semaglutide treatment resulted in lower metabolite concentrations than ForePass, except for alanine, glutamate, glycine, lactate, pyruvate, and citric acid. Smaller differences were obtained instead when Semaglutide treatment was compared to Sham-OP group. Data were scaled to zero mean and unit variance and reported as mean within the three groups. Increase is highlighted in red, decrease in green. *, ^ Mann-Whitney’s test p-value * vs Sham-OP <0.05, ^ vs ForePass < 0.05.

**Figure 2S**

**Metabolic Pathways**

TCA cycle, Tricarboxylic acid cycle; AAA, Aromatic Amino Acids; BCAA, Branched-chain amino acids, CAT, carnitine acetyltransferase; CACT, carnitine acylcarnitine translocase; CPT1, carnitine palmitoyltransferase1; CPT2, carnitine palmitoyltransferase2; PDC, pyruvate dehydrogenase complex.

| **TABLE 1**  **Plasma glucose** |  | **Tukey HSD** | | | |
| --- | --- | --- | --- | --- | --- |
| **TIME** | **Repeated Measure ANOVA** | **p ForePass/Semaglutide** | **p ForePass/sham** | **p Semaglutide/sham** |  |
| 0 | 0.0272 | NS | 0.0220 | NS |  |
| 10 | 0.0026 | 0.0044 | 0.0055 | NS |  |
| 15 | 0.0076 | 0.0276 | 0.0083 | NS |  |
| 20 | 0.0036 | 0.0210 | 0.0034 | NS |  |
| 25 | 0.0058 | NS | 0.0047 | NS |  |
| 30 | 0.0002 | 0.0056 | 0.0002 | NS |  |
| 40 | 0.0021 | 0.0127 | 0.0020 | NS |  |
| 60 | 0.0027 | 0.0182 | 0.0025 | NS |  |
| 80 | 0.0030 | 0.0265 | 0.0025 | NS |  |
| 100 | 0.0063 | 0.0273 | 0.0064 | NS |  |
| 120 | 0.0005 | 0.0045 | 0.0004 | NS |  |
| 140 | 0.0001 | 0.0010 | 0.0002 | NS |  |
| 160 | 0.0014 | 0.0047 | 0.0019 | NS |  |
| 180 | 0.0000 | 0.0000 | 0.0000 | NS |  |

| **Plasma insulin** |  | **Tukey HSD** | | |
| --- | --- | --- | --- | --- |
| **TIME** | **Repeated Measure ANOVA** | **p Forepass/Semaglutide** | **p ForePass/sham** | **p Semaglutide/sham** |
| 0 | 0.0000 | 0.0000 | 0.0000 | NS |
| 10 | 0.0004 | NS | 0.0004 | 0.0032 |
| 15 | 0.0004 | NS | 0.0004 | 0.0040 |
| 20 | 0.0000 | NS | 0.0000 | 0.0001 |
| 25 | 0.0000 | 0.0060 | 0.0000 | 0.0000 |
| 30 | 0.0000 | 0.0001 | 0.0000 | 0.0000 |
| 40 | 0.0000 | 0.0048 | 0.0000 | 0.0000 |
| 60 | 0.0011 | NS | 0.0008 | 0.0424 |
| 80 | 0.0007 | 0.0355 | 0.0005 | 0.0355 |
| 100 | 0.0158 | NS | 0.0136 | NS |
| 120 | 0.0100 | NS | 0.0085 | NS |
| 140 | 0.0039 | NS | 0.0031 | NS |
| 160 | 0.0005 | 0.0253 | 0.0004 | 0.0326 |
| 180 | 0.0003 | 0.0297 | 0.0002 | 0.0110 |

| **Plasma C-peptide** |  | **Tukey HSD** | | | |
| --- | --- | --- | --- | --- | --- |
| **TIME** | **Repeated Measure ANOVA** | **p ForePass/Semaglutide** | **p ForePass/sham** | **p Semaglutide/sham** |  |
| 0 | 0.0168 | NS | 0.0212 | 0.0400 |  |
| 10 | 0.0003 | 0.0179 | 0.0002 | 0.0212 |  |
| 15 | NS | NS | NS | NS |  |
| 20 | 0.0000 | 0.0214 | 0.0000 | 0.0004 |  |
| 25 | 0.0000 | 0.0042 | 0.0000 | 0.0007 |  |
| 30 | NS | NS | NS | NS |  |
| 40 | NS | NS | NS | NS |  |
| 60 | NS | NS | NS | NS |  |
| 80 | NS | NS | NS | NS |  |
| 100 | NS | NS | NS | NS |  |
| 120 | 0.0014 | 0.0013 | NS | 0.0106 |  |
| 140 | NS | NS | NS | NS |  |
| 160 | NS | NS | NS | NS |  |
| 180 | NS | NS | NS | NS |  |
