# Supplementary material for: ForePass outperforms Semaglutide in weight control, glucose metabolism, and gut microbiota in swine
Source: Diabetes Obes Metab. 2025 Sep 30;27(12):7587–601. doi: 10.1111/dom.70167 (PMC12587247; doi:10.1111/dom.70167)
Supplement: Supplementary file 1 — Data S1: Supporting Information [file DOM-27-7587-s001.zip › dom-25-2790-op-File011.pdf]

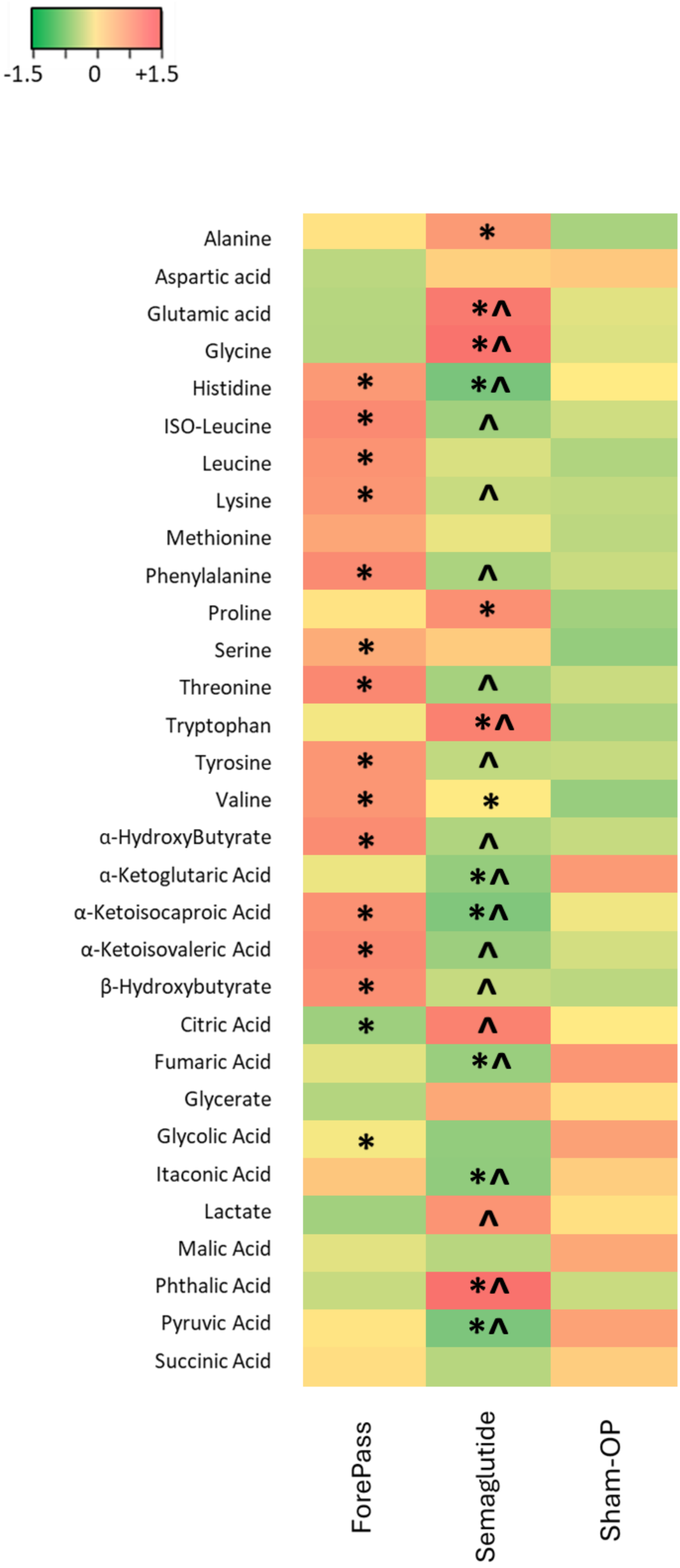

* P-value vs Sham-Op <0.05

^ P-value vs ForePass <0.05
