# Supplementary figures and images for: ForePass outperforms Semaglutide in weight control, glucose metabolism, and gut microbiota in swine
Source: Diabetes Obes Metab. 2025 Sep 30;27(12):7587–601. doi: 10.1111/dom.70167 (PMC12587247; doi:10.1111/dom.70167)

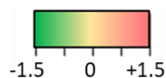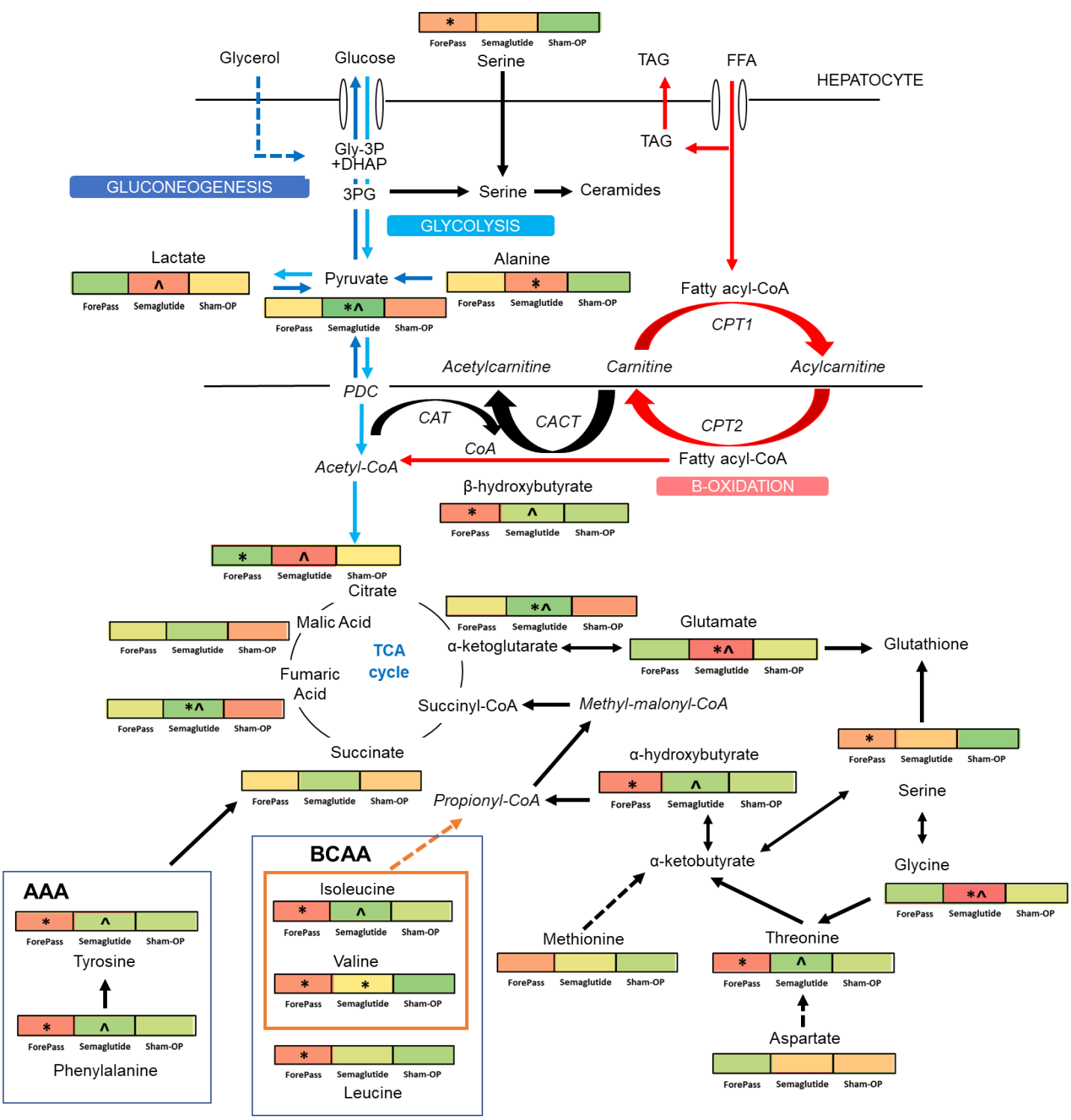

Supplement: Supplementary file 1 — Data S1: Supporting Information [file DOM-27-7587-s001.zip › dom-25-2790-op-File012.pdf]
